# The Acute and Chronic Effects of Dual-Task on the Motor and Cognitive Performances in Athletes: A Systematic Review

**DOI:** 10.3390/ijerph18041732

**Published:** 2021-02-10

**Authors:** Pedro Emílio Drumond Moreira, Gabriel Teles de Oliveira Dieguez, Sarah da Glória Teles Bredt, Gibson Moreira Praça

**Affiliations:** Sports Department, Universidade Federal de Minas Gerais, Belo Horizonte 31270-901, Brazil; pedrodrumondmoreira@hotmail.com (P.E.D.M.); gabrieltelesod@gmail.com (G.T.d.O.D.); sarahtelesb@gmail.com (S.d.G.T.B.)

**Keywords:** sports, working memory, cognition, attention, dual-task, dual-process theory

## Abstract

Athletes must distribute their attention to many relevant cues during a match. Therefore, athletes’ ability to deal with dual-tasks may be different from the non-athlete population, demanding a deeper investigation within the sports domain. This study aimed to systematically review the acute and chronic effects of dual-tasks in motor and cognitive performances in athletes from different modalities. The search for articles followed all the Preferred Reporting Items for Systematic Reviews and Meta-Analyses (PRISMA) guidelines. The keywords used were: “dual-task” OR “double task” OR “multi-task” OR “divided attention” OR “secondary task” OR “second task” AND “working memory” OR “visual” OR “decision making” OR “gaze behavior” OR “attention” AND “sports” OR “athletes” OR “players”. The Scopus, Pubmed, and Web of Science databases were screened for studies comparing single and dual-tasks, in which the participants were athletes competing at any level, and in which at least one of the following variables were investigated: working memory, decision-making, visual search behavior, perception, anticipation, attention, or motor tasks. Articles were screened using pre-defined selection criteria, and methodological quality was assessed by two researchers independently. Following the eligibility criteria, we included 18 articles in the review: 13 on the acute effects, and five on the chronic effects. This review showed that the acute effect of dual-tasks impairs the motor and cognitive performances of athletes (dual-task cost). However, training with dual-tasks (chronic effect) improved working memory skills and attentional control. We conclude that dual-tasks acutely and chronically impacts motor and cognitive performance.

## 1. Introduction

In open sports such as soccer, basketball, and handball, players’ ability to distribute their attention between different relevant cues in the environment (e.g., ball, opponents, and teammates) is mandatory for successful decision-making [1]. Simultaneously, players must execute various motor actions, such as running, passing, and dribbling, characterizing a dual-task demand in team sports [2].

The dual-process theory explains people’s ability to perform multiple tasks at a time and suggests that human behavior is controlled by two different information processing systems: automatic and controlled [3]. Automatic processing (type 1) is fast and autonomous, activated by triggers; for example, when one is questioned “What is your name?”, he/she will provide an automatic fast answer. Controlled processing (type 2) is slow and activates the working memory (WM), requiring information storage and processing, and attentional control (the latter is required in both automatic and controlled systems) [4]. For example, in a soccer match, after receiving the ball, the player realizes that a teammate is in a good position and, hence, decides to pass the ball to him, which is done with low attentional resources allocated in the motor execution (type 1 processing). Simultaneously, attentional resources are allocated to identify and interpret the opponents’ positioning on the pitch (type 2 processing). These multiple tasks executed simultaneously are known in the scientific literature as dual-tasks.

Previous studies in different areas investigated the acute effects of dual-task practicing on injury prevention (e.g., posture and balance) and motor enhancement (e.g., gaiting, walking, and running) in young healthy individuals [5], children [6,7], elderly people [8], patients with Parkinson and Alzheimer diseases [9], and patients with brain injuries [10]. In these studies, dual-tasks were used to identify participants’ motor and cognitive capabilities by combining motor and cognitive demands to overload the working memory [11]. In general, they observed a reduction in performance in the dual-task compared to the control condition (single-task), in both normal and impaired individuals. The dual-task cost paradigm explains these results, suggesting that cognitive tasks often require most of the attentional resources, overloading the working memory [12] and, hence, reducing cognitive and motor performance. However, studies on the chronic effect of dual-task practicing showed increases in motor and cognitive performance, even in impaired individuals [13,14], probably due to improved attention recruitment related to a larger capacity of the working memory. Nevertheless, there were no recent attempts to summarize the available literature on this topic, which limits the whole comprehension of the phenomenon. Most of the available literature reviews on this topic were published before 2014 [15,16,17,18], and the population was not always composed of athletes, which reinforces the need for the current review to summarize recent findings within this topic.

In open sports, the execution of dual-tasks occurs in a highly time-constrained environment, which might also lead to a choking condition that impairs performance [19,20]. Due to the characteristics of the action during different sports, the use of dual-tasks during training might be useful for reproducing sport demands by simultaneously requiring the perception of relevant cues and the execution of technical actions [2]. Previous studies on dual-tasks in sport showed methodological differences related to the task characteristics, the dual-task cost, the participants, and the study design, since studies have not used dual-tasks during the whole training session [12,21] that complicate the comprehension of the acute and chronic effects of dual-tasks on cognitive and motor performances. Besides, the different characteristics between sports (e.g., individual or team, and open or closed) make it difficult to establish solid conclusions on this topic. We could expect that the results reported for non-athletes would apply to the sports domain since the cognitive processes that support dual-task performance are present in both contexts [22,23], although no previous study has attempted to summarize the available literature on this topic.

Based on the abovementioned issues, this study aimed to systematically review the acute and chronic effects of dual-task practicing on the cognitive and motor performances of athletes. 

## 2. Materials and Methods

This systematic review followed all of the Preferred Reporting Items for Systematic Reviews and Meta-Analyses (PRISMA) guidelines [24]. A meta-analysis was not conducted because of the high measurement and methodological heterogeneity observed in the selected studies.

### 2.1. Information Sources

The search for relevant articles was conducted in May 2020 from three different databases, Scopus, Pubmed, and Web of Science, considering articles published between January 2015 and May 2020. The following keywords were included in the search: “dual-task” OR “double task” OR “multi-task” OR “divided attention” OR “secondary task” OR “second task” AND “working memory” OR “visual” OR “decision making” OR “gaze behavior” OR “attention” AND “sports” OR “athletes” OR “players”. These keywords were selected based on the authors’ experience in this topic, to adequately search for links between dual-task and cognitive/motor performances. The keyword “working memory” was selected because of previous studies pointing out the high relevance of this cognitive process to understand the underlying mechanisms of dual-task cost.

### 2.2. Eligibility Criteria

The articles found in the search were included in the review if they met the following criteria: (a) studies comparing single and dual-tasks (studies on only one type of task were excluded); (b) participants were athletes competing at any level (no age or sex restrictions were made to the population of the selected studies to possibility a higher generalization of the results); (c) studies were original, reviews were excluded; (d) studies in which at least one of the following variables was reported: working memory, decision-making, visual search behavior, perception, anticipation, attention, or motor tasks; (e) studies published in English; (f) studies published in 2015 or later (last six years); and (g) published in peer-review journals. Unpublished studies were not included in the review for two main reasons. Firstly, they could result in redundancy, as many theses and dissertations (a significant part of the unpublished materials) are later published as articles. Secondly, the absence of a peer-review process would allow the inclusion of low methodological quality studies. Concerning the period of the review, we decided to include only recent articles (last six years, from January 2015 to May 2020) to reduce the methodological heterogeneity of the selected studies, since many of the current ones use tests and procedures recently proposed and validated (for example, 3D-motion [21,25,26]). Additionally, reviews on this topic were found in 2014, which would lead us to replicate the findings of previous reviews [15,16,17,18].

### 2.3. Study Selection and Data Extraction

Two independent researchers (PM and GT) selected the articles based on the titles and abstracts. The selected articles were then exported from the database in BibTeX format and saved in the Mendeley Reference Management Software and Research Network [27], which was used to remove the duplicates. The remaining articles were fully analyzed for eligibility. When the researchers disagreed on the inclusion of an article, a third researcher, with more than five years of experience in publishing scientific articles, was consulted (GP). This procedure was adopted to ensure a reduced bias in selecting the articles and was previously adopted in the literature [28,29].

**Figure 1:**
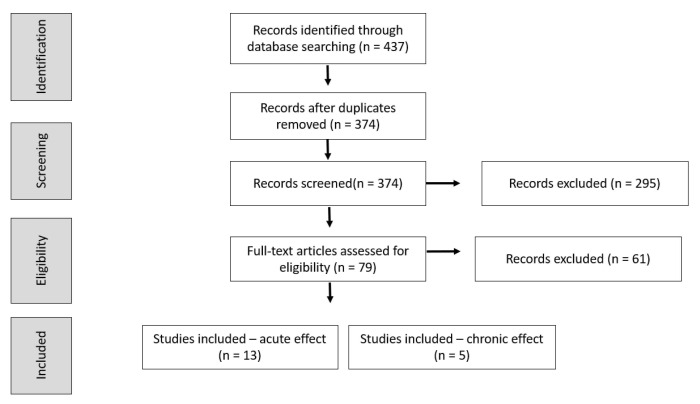
Flowchart of the systematic search for articles.

Figure 1 shows the selection process of the articles. Mainly excluding reasons included the articles not showing comparison between single and dual-tasks, having participants being impaired individuals, and having dependent variables not in the scope of the present review. After analyzing all the inclusion criteria, 18 articles were selected for the quantitative and qualitative synthesis: 13 on the acute, and 5 on the chronic effects of dual-tasks. In the identification step, 63 duplicates were removed. Next, 374 were screened by evaluating titles and abstracts, resulting in the exclusion of 295 articles. The main reasons for exclusion were due to sample characteristics (studies not focused on athletes), dependent variables (not those analyzed in the current review), and the absence of dual-tasks in the procedure. Finally, the full texts of 79 articles were analyzed for the eligibility criteria, resulting in 61 exclusions. Articles were mainly excluded because of the absence of dual-tasks in the experimental design.

### 2.4. Data Items

The following information was extracted from each included article: authors, title, participants (number, gender, age, and competitive level), sport, task characteristics, dependent and independent variables, average values for all dependent variables, and main results. We also estimated the dual-task cost based on the Beurskens and Bock [30] protocol to compare performance between single and dual-tasks using the formula ((DT − ST)/ST × 100), in which ST represents the single-task performance and DT represents the dual-task performance. Studies were classified as chronic if the measures were taken pre- and post- an intervention program, clearly introduced by the researchers. As there is no definite information about the minimum training period for physical or cognitive adaptations to dual-task training, there was no minimum cut-off for the training length. On the other hand, if single measures were obtained and there was no intervention program being tested, the studies were classified as acute.

### 2.5. Risk of Bias in Individual Studies

Previous systematic reviews opted to analyze the methodological quality of the studies, as this would reduce the risk of bias when interpreting the results [31]. For this reason, the methodological quality of the selected studies was evaluated by two independent researchers (PM and GP) using a modified version of the Quality Index Scale [32], recently adopted in systematic reviews in sport [31] and including 14 out of the 24 original items. The following items were analyzed. Item 1: Is the hypothesis/aim/objective of the study clearly described?; Item 2: Are the main outcomes to be measured clearly described in the Introduction or Methods sections?; Item 3: Are the characteristics of the patients included in the study clearly described?; Item 6: Are the main findings of the study clearly described?; Item 7: Does the study provide estimates of the random variability in the data for the main outcomes?; Item 10: Have actual probability values been reported (e.g., 0.035 rather than <0.05) for the main outcomes, except where the probability value is less than 0.001?; Item 12: Were those subjects who were prepared to participate representative of the entire population from which they were recruited?; Item 15: Was an attempt made to blind those measuring the main outcomes of the intervention?; Item 16: If any of the results of the study were based on “data dredging”, was this made clear?; Item 18: Were the statistical tests used to assess the main outcomes appropriate?; Item 20: Were the main outcome measures used accurate (valid and reliable)?; Item 22: Were study subjects in different intervention groups (trials and cohort studies), or were the cases and controls (case-control studies) recruited over the same period?; Item 23: Were study subjects randomized to intervention groups?; Item 25: Was there an adequate adjustment for confounding in the analyses from which the main findings were drawn? [32]. Table 1 shows the results of the assessment of the methodological quality of each study included in the review.

## 3. Results

Table 1 shows the methodological quality of the selected studies. Within the acute studies, the lowest reported score was 0.75, while the highest value was 1.00. All the studies presented high methodological quality. Similarly, concerning the studies on the chronic effects, the lowest reported value was 0.8, while the highest score was 1.00, which denotes a high methodological quality of the articles.

Table 2 shows the main characteristics of the selected studies on the acute effect of dual-tasks. Most of the studies included heterogeneous participants (both men and women, or athletes of different levels of expertise) and analyzed different sports (beach volleyball, basketball, hockey, table tennis, cricket, soccer, American football, cross country, powerlifting, baseball, cheerleading, fencing, handball, and boxing). Various stimuli were observed in secondary tasks (auditory, visual, memorizing, mathematical operations, and balance). Twelve of the thirteen studies had a cost for dual-tasks, that is, the performance in motor and cognitive tasks was inferior in dual compared with single-tasks. Only one study showed a different result [41]. In studies that compared athletes of different levels (e.g., skilled vs. less-skilled, and elite vs. intermediate), higher-level athletes had a lower cost of performance for dual-tasks than lower-level athletes.

Table 3 shows the main characteristics of the selected studies on the chronic effect of dual-tasks practicing. Similar to the studies on the acute effects, three of the five studies included both men and women. Only one of the five studies included high-level athletes; the others analyzed amateurs. The investigated sports were beach volleyball, tennis, football, hockey, and badminton. Four of the five selected studies presented a virtual task (3D Motion) for training, which required athletes to track multiple objects, implying attentional distribution. In all studies, the groups that practiced with dual-tasks improved both motor and cognitive performances after the training sessions.

## 4. Discussion

Previous studies out of the sports context showed that dual-tasks lead to acute impairments in the performance of both primary and secondary tasks, but can lead to improvements over time [5,14]. Given the motor and cognitive requirements of the sport, we expected to find the same acute and chronic effects in the sports context. The findings of the present review indicated that the acute exposure to dual-tasks impairs the performance in both motor and cognitive tasks, and that chronic exposure to dual-tasks (i.e., training) improves both performances.

As we hypothesized, even though athletes are familiar with time-constrained situations and multitasking, there was a cost in motor and cognitive performances for the execution of dual-tasks. Concerning the cost of cognitive performance, some studies suggested that the demand imposed by secondary tasks exceeded athletes’ capacity to manage information leading to impaired performance. For example, Laurin and Finez [12] observed that the higher the level of difficulty of secondary tasks (multiplication > subtraction > sum), the higher the cost in performance. Given the close relationship between working-memory capacity and attentional control [47], we suggest that individuals with high working-memory capacity could optimize attentional resources for solving a cognitive task while performing the motor task. This rationale can be supported by the studies that compared experts and novices and found a lower dual-task cost in the experts, reinforcing the importance of a high working-memory capacity (observed in the experts) in the processes of attentional control [47,48,49]. These studies hypothesized that the high demand for dual-tasks interfered with the working-memory capacity and, consequently, in the attentional control, made it harder for athletes to extract relevant clues in the environment for the tactical decision, mainly for individuals with low working-memory capacity. However, the relationship between dual-tasks performance, visual search behavior, and attentional control remains unclear; future studies are recommended to elucidate how and to where athletes direct their attentional focus (e.g., gaze behavior) in situations with cognitive interference (dual-tasks). This information will allow coaches to direct athletes’ attention to the most relevant cues during tactical training.

The attentional focus may also explain the drop in motor performance in dual-tasks. Previous research has shown that the difficulty of the dual-tasks led individuals to the state of “choking” [19], with increased levels of anxiety and the explicit direction of the attentional focus to the execution of the movement (autofocus). Explicit monitoring of movement execution demands the working memory to process movement information (type 2 processing, a slow process) differently from the fast movement responses required from the athletes in competitive sport contexts [50]. This rationale reinforces the importance of dual-tasks training in open sports to decrease the need for controlled processing and drops in the motor performance during competition.

Concerning the chronic effects, training with dual-tasks improved both motor and cognitive performance. Bherer et al. [51] suggested that dual-tasks training leads to the development of new perceptual strategies (optimizing attentional focus for the relevant clues in the task) that contribute to improved decision-making [52], as supported by some findings of the present review [21,26]. The significant increase in the duration of the quiet eye [45] after training with dual-tasks indicate that the athletes directed their attention to specific spots for a longer time to extract relevant information for the tactical decision. Additionally, the improvement in the efficiency of attentional control after training can be supported by other enhanced cognitive processes such as the ability to sustain attention and processing speed, observed in the study of Fleddermann et al. [21].

Despite the innovative information on the acute and chronic effects of dual-tasks in sports, the present study has some limitations. Although most of the studies presented high methodological quality, some intervening variables were not controlled. For example, there seemed to be differences in the cost of dual-tasks between athletes of different levels (experts, non-experts, and novices), different sexes, and from the team or individual sports. Calculating these differences was not possible due to the heterogeneity of participants within each study. Besides, the various methodological procedures (e.g., type of stimulus, training time, and dependent and independent variables) limited the conclusions; therefore, investigating the dual-task paradigm in sport is still recommended. At this point, future studies should attempt to conduct meta-analysis when the heterogeneity of the methods and variables are reduced, providing definitive pieces of information on this phenomenon. Although the studies included in this review investigated athletes, the experiments were mostly laboratory and the tasks were not representative of the investigated sports; for example, technical performance and reaction time were assessed in closed scenarios, which are sensitively different from the tactical-technical context in which movements are required in sports. Finally, we analyzed articles published only in the six years. This was needed to reduce the methodological heterogeneity of the observed studies, as different procedures were recently proposed in the literature and adopted by the researchers. However, we are aware that relevant but older studies were not selected, and this is a possible limitation.

Given these aspects, it is a challenge for future research to investigate the effects of dual-tasks in situations similar to sports, that is, that couple the technical and tactical components (e.g., small-sided games). For example, we recommend including dual-tasks in game-based tasks and investigating how the tactical and technical performances are impaired. Additionally, including dual-tasks in both game-based and laboratory techniques during a training period in team sports and observing the long-term development of tactical skills would help to understand the links between working memory, attentional focus, and tactical knowledge development. The knowledge about the perceptual-cognitive processes (attention, gaze behavior, and working memory) that are manifested in dual-tasks within real contexts is relevant for coaches to guide the teaching-learning process.

## 5. Conclusions

Based on the results of this review, we concluded that athletes experience a decrease in motor and cognitive performances in dual-tasks. However, training with dual-tasks seems to favor the improvement of the working memory capacity and, consequently, the attentional control that is related to perception. These results can help coaches to plan dual-tasks during training to optimize athletes’ motor and cognitive performances.

## Figures and Tables

**Table 1:** The methodological quality of each study included in the review based on the Quality Index Scale and divided according to study design (acute and chronic effects).

Item Code	1	2	3	6	7	10	12	15	16	18	20	22	23	25	Final Score (14 Items)
Study	Acute Effect														
Cochrane et al. [33]	1	1	1	1	1	0	1	U	1	1	0	1	U	1	0.83
Fleddermann and Zentgraf [34]	1	1	1	1	1	1	0	U	1	1	1	1	U	1	0.92
Gutierrez-Davila et al. [35]	1	1	1	1	1	0	0	U	1	1	0	1	U	1	0.75
Helm et al. [36]	1	1	1	1	1	1	0	U	1	1	0	1	U	1	0.83
Howell et al. [37]	1	1	1	0	1	1	1	U	1	1	1	0	U	1	0.83
Howell et al. [38]	1	0	1	1	1	1	1	U	1	1	1	1	U	0	0.83
Laurin and Finez [12]	1	1	1	1	0	1	1	U	1	0	0	1	U	1	0.75
Lynall et al. [39]	1	1	1	1	1	1	0	U	1	1	1	1	U	1	0.92
Qiu et al. [40]	1	1	1	1	1	1	0	U	1	1	1	1	U	1	0.92
Runswick et al. [41]	1	1	1	1	1	1	0	U	1	1	1	1	1	1	0.92
Schaefer and Scornaienchi [42]	1	1	1	1	0	1	1	U	1	1	1	1	U	1	0.92
Tapper et al. [43]	1	1	1	1	1	1	0	U	1	1	1	1	U	1	0.92
Van Biesen et al. [44]	1	1	1	1	1	1	1	U	1	1	1	1	U	1	1
Chronic Effect															
Ducrocq et al. [45]	1	1	1	1	1	1	1	U	1	1	1	1	1	1	1
Fleddermann et al. [21]	1	1	1	1	1	1	1	U	1	1	1	1	U	1	1
Harris et al. [46]	1	1	1	1	1	1	1	U	1	1	1	1	U	1	1
Romeas et al. [25]	1	1	1	1	0	1	0	U	1	1	U	U	U	1	0.8
Romeas et al. [26]	1	1	1	1	0	1	0	U	1	1	U	U	U	1	0.8

**Table 2:** Selected studies on the acute effect of dual-tasks.

Authors	Participants	Age	Competitive Level	Tasks	Dependent Variables	Results	Dual-Task Cost (DT − ST)/ST × 100
Fleddermann and Zentgraf [34]	24 beach volleyball players (21 women and 3 men)	Mean age = 19.2 years, SD = ±4.2	National	ST: volleyball block DT: volleyball block + visual stimulus	Decision-making, jump height, and stride length	Jump height = ST > DT Stride length = ST > DT Decision-making (error) = ST < DT	Jump Height = 4.13% Stride length = 37.6%
Qiu et al. [40]	44 male basketball players	Elite: Mean age = 20.68 SD = ±1.39 years Intermediate: mean age = 20.20 SD = ±2.35 years	National and College	ST: accuracy in the multiple object tracking task with two distractors DT: accuracy in the location in the multiple object tracking task with four distractors	Accuracy in the multiple object tracking task	Accuracy: Elite = ST > DT Intermediate = ST > DT	Elite = 36.4% Intermediate = 54.2%
Tapper et al. [43]	11 Hockey players (4 men and 7 women)	Women: Mean age = 18.7 SD = ±1.2 Men: Mean age = 22.2 SD = ±0.9	College	ST: accuracy in the multiple object tracking task DT: accuracy in the multiple object tracking task + task with an acoustic requirement	Accuracy in the multiple object tracking task	Accuracy: Men = ST > DT Women = ST > DT	Men = 12.3% Women = 11%
Schaefer and Scornaienchi [42]	22 table tennis players (7 women and 15 men, 11 experts and 11 non- experts)	Experts: Mean age = 25.5 SD = ±2.6 Non- experts: Mean age = 23.6 SD = ±2.2	Not mentioned	ST: technical-tactical accuracy DT: technical-tactical accuracy + working memory task (3-back stimuli test)	Tecnhical-tactical accuracy; working memory capacity	Technical-tactical accuracy: experts = ST > DT, non-expert = ST > DT; Working memory capacity: experts = ST > DT, non-experts = ST > DT	Working memory capacity: Expert = 10% Non-expert = between 30% and 50% Technical-tactical performance: Expert = 10% Non-expert = 30%
Van Biesen et al. [44]	103 participants from various sports (70 men and 33 women)	Men: Mean age = 21.4 SD = ±2.6 Women: Mean age = 20.5 SD = ±1.9	Amateur	ST: accuracy in the multiple object tracking task DT: accuracy in the multiple object tracking task + balance task	Accuracy in the multiple object Tracking task; Static balance control performance	Accuracy in the decision task: ST > DT Performance in the balance task: ST > DT, effect size	Static balance control task: 12.89% Multiple object tracking task = 1.34%
Runswick et al. [41]	18 male cricket players (9 skilled and 9 less-skilled)	Skilled: Mean age 22.6 SD = ±7.8 years Less-skilled: Mean age = 28.9 SD = ±6.7 years	Regional and International	ST: anticipation task DT: anticipation task + working memory task (7-back stimuli)	Anticipation accuracy and working memory capacity	Accuracy in anticipation: Skilled = DT > ST Less-skilled = DT > ST Working memory capacity: Skilled = DT > ST; Less-skilled = DT > ST	Accuracy in anticipation: Skilled -29% Less-skilled -16% Working memory capacity: No information for calculation
Cochrane et al. [33]	87 participants from various sports (39 women and 48 men)	Mean age = 20.6 SD = ±1.8 years	College	ST: simple visual stimuli for decision DT: simple visual stimuli for decision + visual task	Reaction time	Reaction time = DT > ST	Reaction time = 73%
Gutierrez-Davila et al. [35]	25 fencing players (15 men and 10 women)	Homens: Mean age = 21.1 SD = ±4.9 years Mulheres: Mean age = 21.4 SD = ±2.3 years	Elite	ST: attacking actions against an opponent after a pre-established visual stimulus DT: an attentional task in which players were required to react differently to visual stimuli in the trunk and the head.	Reaction time; Speed in the attacking actions; Technical-tactical offensive and defensive performance	Reaction time = DT > ST Speed of attack actions = ST > DT Technical-tactical defensive performance = ST > DT	Reaction time = 33% Attacking speed = 7% Technical-tactical performance = 103%
Helm et al. [36]	33 male handball goalkeepers (15 elite and 18 amateurs)	Mean age = 24.4 years SD = ±4.9	Elite and amateur	ST: goalkeeping with one ball DT: goalkeeping with two balls	Reaction time	Reaction time: elite and amateurs = DT > ST	Reaction time: elite 20% amateurs: 17%
Laurin and Finez [12]	90 male soccer players	Study 1: Mean age = 19.2 SD = ±1.3 Study 2: Mean age = 19.2 SD = ±1.1 Study 3: Mean age = 19.9 SD = ±1.3	College	ST: juggling performance DT: juggling performance + perform arithmetic subtraction operations + count down from 3 by 3 from 300 juggling performance + multiplication task	Performance in juggling performance	Technical performance = ST > DT	Juggling performance + Subtraction of numbers = 17% Juggling performance + 2 in 2 counting = 13% Juggling performance + multiplication = 26.5%
Lynall et al. [39]	15 participants from various sports (6 men and 9 women)	Mean age = 19.7 SD = ±1.6 years	College	ST: balance task DT: balance task + Brooks visuospatial task	Path Length Speed Velocity Working memory capacity	Path Length = ST > DT Speed = ST > DT Velocity = ST > DT Working memory capacity = ST = DT (no significant difference)	Path Length = 7.6% Speed = 4% Velocity = 28%
Howell et al. [37]	61 female boxing figthers	Median = 27 Range = 21–36 years	Elite	ST: (timed-up and-go test) DT: (timed-up-and-go test) + Number counting task The tasks were applied in two moments (pre-tournament and post- tournament)	Time to complete the tasks	Pre-tournament-ST = 8.7 (6.3–13.7) DT = 11.7 (6.7–21.7) Post-tournament-ST = 8.3 (6.7–15.0) DT = 10.9 (8.0–19.7)	Pre-tournament task execution time = 34.5% Post-tournament = 31.3%
Howell et al. [38]	31 participants from various sports (13 men and 18 women)	Mean age = 14.9 SD = ±1.8	Not reported	ST: walk test DT: walk test + cognitive task (spell a 5-letter word backwards, subtract a two-digit number from 6 or 7 and recite the months in reverse order)	Gait speed, stride length, cadence (steps/min), double support time and accuracy in the cognitive task	Gait speed, stride length, cadence and double support time = ST > DT; Cognitive task = ST = DT	Walking speed = 99.4% Stride lengths = 26.8% Cadence = 30.6% Double support time = 28.8%

ST: Single Task; DT: Dual-Task.

**Table 3:** Selected studies on the chronic effect of dual-tasks.

Authors	Participants	Age	Competitive Level	Dependent Variables	Dual-Tasks Training	Results
Ducrocq et al. [45]	30 amateur table tennis players (25 men and 5 women).	Mean age = 33 years Range = 17–50 Control group: Mean age = 32.46 SD = ±13.60 Training group: Mean age = 34.76 SD = ±13.29	Amateur	Working memory (near task) effectiveness of technical-tactical actions in the tennis task (far task); Quiet eye period; Quiet eye onset and offset.	DT: computer tasks to memorize a visual stimulus + task to memorize an auditory stimulus (letters), simultaneously. Training regimen: 20 blocks of 20 + n (number of letters) attempts. Each training session lasted approximately 30 min and was performed in 10 days.	Working memory: increased score after training Effectiveness of technical-tactical action in the tennis task (far task): increased accuracy after training Quiet Eye Offset: increased fixation after training Quiet Eye Period and onset: no significant differences after training
Fleddermann et al. [21]	43 beach volleyball players, 22 in the intervention group (2 men and 20 women) and 21 in the active control group (5 men and 16 women).	Intervention group: Mean age = 16.38 SD = ±1.7 Control group: Mean age = 21.38 SD = ±4.53	Elite	Working memory capacity; Jump height in a specific task (beach volleyball); Accuracy in 3D Motion task; Attentional capacity; Processing speed	DT: the specific or nonspecific motor task of volleyball + 3D Motion task. Training regimen: Eight weeks with two blocks per week, lasting 30 min per session. Each block comprised three sessions, eight minutes each with a three-minute break in-between.	Performance in the 3D motion task: training group showed higher scores compared with the control group in the post-test Sustained attention: training group showed higher scores compared with the control group in the post-test Processing speed: training group showed higher scores compared with the control group in the post-test Jump height: performance in single tasks was higher than in dual-tasks in the post-test Working memory capacity: no significant difference between groups and time.
Harris et al. [46]	36 hockey and soccer players (22 women and 14 men).	Mean age = 22.5 years SD = ±3.7	Amateur	Working memory capacity; Accuracy in the 3D Motion test (object tracking) Visual search behavior (centroid and amplitude)	3D Motion Training: Multi-object tracking Training regimen: Each session consisted of four blocks of 20 objects for tracking. Each session lasted 20 min. The training group returned for another 20 min of training after 12–14 days.	Accuracy in the 3D Motion task: training group showed a higher score compared with the control group in the post-test Working memory capacity: training group showed a higher score compared with the control group in the post-test Visual behavior: no significant difference between groups.
Romeas et al. [25]	23 male soccer players.	Training group (3D Motion): Mean age = 21.27 SD = ±0.81 Active control: Mean age = 21.39 SD = ±1.03 Passive control: Mean age = 22.48 SD = ±0.71	Amateur	Decision-making performance in small-sided games and passing, dribbling and shooting performance	3D Motion Training: football scenes + decision-making responses. Training regime: two sessions per week over 5 consecutive weeks. Athletes participated in at least 3 out of the 5 sessions of 3D Motion	Only the training group improved the passing decision-making from pre- to post-test. There were no differences in kicking and dribbling.
Romeas et al. [26]	29 badminton players (6 women and 23 men).	Mean age = 22.98 SD = ±2.77 years	Amateur	Speed, reaction time, decision making	Training: 3D Motion training + motor decision-making task and training com 3D Motion + perceptual decision-making task. Training regimen: nine 30-min sessions	The group that trained to combine 3D Motion task + motor decision-making task showed better performance in decision making and reaction time in the post-test moment. The group that trained to combine 3D Motion task + perceptual decision-making task did not improve performance in any of the variables.

DT: Dual-Task.

## Data Availability

The data presented in this study are available on request from the corresponding author.

## References

[1] Williams A.M., Davids K., Williams J.G.P. (1999). Visual Perception and Action in Sport.

[2] Casanova F., Oliveira J., Williams M., Garganta J. (2009). Expertise and perceptual-cognitive performance in soccer: A review. Rev. Port. Ciências Desporto.

[3] Evans J.S.B.T., Stanovich K.E. (2013). Dual-Process Theories of Higher Cognition: Advancing the Debate. Perspect. Psychol. Sci..

[4] Baddeley A. (2003). Working memory: Looking back and looking forward. Nat. Rev. Neurosci..

[5] Beurskens R., Steinberg F., Antoniewicz F., Wolff W., Granacher U. (2016). Neural Correlates of Dual-Task Walking: Effects of Cognitive versus Motor Interference in Young Adults. Neural Plast..

[6] Beurskens R., Muehlbauer T., Grabow L., Kliegl R., Granacher U. (2016). Effects of Backpack Carriage on Dual-Task Performance in Children During Standing and Walking. J. Mot. Behav..

[7] Beurskens R., Muehlbauer T., Granacher U. (2015). Association of dual-task walking performance and leg muscle quality in healthy children. BMC Pediatrics.

[8] Plummer P., Zukowski L.A., Giuliani C., Hall A.M., Zurakowski D. (2015). Effects of Physical Exercise Interventions on Gait-Related Dual-Task Interference in Older Adults: A Systematic Review and Meta-Analysis. Gerontology.

[9] Belghali M., Chastan N., Davenne D., Decker L.M. (2017). Improving Dual-Task Walking Paradigms to Detect Prodromal Parkinson’s and Alzheimer’s Diseases. Front. Neurol..

[10] Solomito M.J., Kostyun R.O., Wu Y.H., Mueske N.M., Wren T.A.L., Chou L.S., Ounpuu S. (2018). Motion analysis evaluation of adolescent athletes during dual-task walking following a concussion: A multicenter study. Gait Posture.

[11] Rhodes S., Jaroslawska A.J., Doherty J.M., Belletier C., Naveh-Benjamin M., Cowan N., Camos V., Barrouillet P., Logie R.H. (2019). Storage and Processing in Working Memory: Assessing Dual-Task Performance and Task Prioritization Across the Adult Lifespan. J. Exp. Psychol..

[12] Laurin R., Finez L. (2020). Working memory capacity does not always promote dual-task motor performance: The case of juggling in soccer. Scand. J. Psychol..

[13] Lüder B., Kiss R., Granacher U., Lueder B., Kiss R., Granacher U. (2018). Single- and dual-task balance training are equally effective in youth. Front. Psychol..

[14] Agmon M., Kelly V.E., Logsdon R.G., Nguyen H., Belza B. (2015). The effects of Enhancefitness (EF) training on dual-task walking in older Adults. J. Appl. Gerontol..

[15] Wollesen B., Voelcker-Rehage C. (2014). Training effects on motor-cognitive dual-task performance in older adults: A systematic review. Eur. Rev. Aging Phys. Act..

[16] Schoene D., Valenzuela T., Lord S.R., De Bruin E.D. (2014). The effect of interactive cognitive-motor training in reducing fall risk in older people: A systematic review. BMC Geriatr..

[17] Strobach T., Salminen T., Karbach J., Schubert T. (2014). Practice-related optimization and transfer of executive functions: A general review and a specific realization of their mechanisms in dual tasks. Psychol. Res..

[18] Schaefer S. (2014). The ecological approach to cognitive-motor dual-tasking: Findings on the effects of expertise and age. Front. Psychol..

[19] Baumeister R.F. (1984). Choking under pressure: Self-consciousness and paradoxical effects of incentives on skillful performance. J. Personal. Soc. Psychol..

[20] Wilson M.R., Wood G., Vine S.J. (2009). Anxiety, attentional control, and performance impairment in penalty kicks. J. Sport Exerc. Psychol..

[21] Fleddermann M.-T.T., Heppe H., Zentgraf K. (2019). Off-Court Generic Perceptual-Cognitive Training in Elite Volleyball Athletes: Task-Specific Effects and Levels of Transfer. Front. Psychol..

[22] Furley P., Wood G. (2016). Working Memory, Attentional Control, and Expertise in Sports: A Review of Current Literature and Directions for Future Research. J. Appl. Res. Mem. Cogn..

[23] Weigel P., Raab M., Wollny R. (2015). Tactical Decision Making in Team Sports—A Model of Cognitive Processes. Int. J. Sport. Sci..

[24] Moher D., Liberati A., Tetzlaff J., Altman D.G., Group T.P. (2009). Preferred Reporting Items for Systematic Reviews and Meta-Analyses: The PRISMA Statement. Ann. Intern. Med..

[25] Romeas T., Guldner A., Faubert J. (2016). 3D-Multiple Object Tracking training task improves passing decision-making accuracy in soccer players. Psychol. Sport Exerc..

[26] Romeas T., Chaumillon R., Labbé D., Faubert J. (2019). Combining 3D-MOT With Sport Decision-Making for Perceptual-Cognitive Training in Virtual Reality. Percept. Mot. Ski..

[27] Singh J. (2010). Mendeley: A free research management tool for desktop and web. J. Pharmacol. Pharmacother..

[28] Sarmento H., Marques A., Field A., Martins J., Gouveia É.R., Laura P.M., Nestor O.S., Rodríguez D.A., Clemente F.M. (2020). Genetic influence on football performance: A systematic review. Hum. Mov..

[29] Afonso J., Bessa C., Nikolaidis P.T., Teoldo I., Clemente F. (2020). A systematic review of research on Tactical Periodization: Absence of empirical data, burden of proof, and benefit of doubt. Hum. Mov..

[30] Beurskens R., Bock O. (2012). Age-Related Deficits ofDual-TaskWalking: A Review. Neural Plast..

[31] Bujalance-Moreno P., Latorre-Román P.Á., García-Pinillos F. (2019). A systematic review on small-sided games in football players: Acute and chronic adaptations. J. Sports Sci..

[32] Downs S.H., Black N. (1998). The feasibility of creating a checklist for the assessment of the methodological quality both of randomised and non-randomised studies of health care interventions. J. Epidemiol. Community Health.

[33] Cochrane G.D., Christy J.B., Almutairi A., Busettini C., Swanson M.W., Weise K.K. (2019). Visuo-oculomotor Function and Reaction Times in Athletes with and without Concussion. Optom. Vis. Sci..

[34] Fleddermann M.-T., Zentgraf K. (2018). Tapping the Full Potential? Jumping Performance of Volleyball Athletes in Game-Like Situations. Front. Psychol..

[35] Gutiérrez-Davila M., Rojas F.J., Gutiérrez-Cruz C., Navarro E., Gutierrez-Davila M., Javier Rojas F., Gutierrez-Cruz C., Navarro E. (2017). Effect of dual-attention task on attack and defensive actions in fencing. Eur. J. Sport Sci..

[36] Helm F., Reiser M., Munzert J. (2016). Domain-Specific and Unspecific Reaction Times in Experienced Team Handball Goalkeepers and Novices. Front. Psychol..

[37] Howell D.R., O’Brien M.J., Raghuram A., Shah A.S., Meehan W.P. (2018). Near Point of Convergence and Gait Deficits in Adolescents after Sport-Related Concussion. Clin. J. Sport Med..

[38] Howell D.R., Meehan W.P., Loosemore M.P., Cummiskey J., von Rosenberg J.-P.G., McDonagh D. (2017). Neurological tests improve after Olympic-style boxing bouts: A pretournament and post-tournament study in the 2016 Women’s World Boxing Championships. Br. J. Sports Med..

[39] Lynall R.C., Blackburn J.T., Guskiewicz K.M., Marshall S.W., Plummer P., Mihalik J.P. (2019). Functional balance assessment in recreational college-aged individuals with a concussion history. J. Sci. Med. Sport.

[40] Qiu F., Pi Y., Liu K., Li X., Zhang J., Wu Y. (2018). Influence of sports expertise level on attention in multiple object tracking. PeerJ.

[41] Runswick O.R., Roca A., Mark Williams A., Bezodis N.E., Mcrobert A.P., North J.S. (2018). The impact of contextual information and a secondary task on anticipation performance: An interpretation using cognitive load theory. Appl. Cogn. Psychol..

[42] Schaefer S., Scornaienchi D. (2020). Table Tennis Experts Outperform Novices in a Demanding Cognitive-Motor Dual-Task Situation. J. Mot. Behav..

[43] Tapper A., Gonzalez D., Roy E., Niechwiej-Szwedo E. (2017). Executive function deficits in team sport athletes with a history of concussion revealed by a visual-auditory dual task paradigm. J. Sports Sci..

[44] Van Biesen D., Jacobs L., McCulloch K., Janssens L., Vanlandewijck Y.C. (2018). Cognitive-motor dual-task ability of athletes with and without intellectual impairment. J. Sports Sci..

[45] Ducrocq E., Wilson M., Smith T.J., Derakshan N. (2017). Adaptive Working Memory Training Reduces the Negative Impact of Anxiety on Competitive Motor Performance. J. Sport Exerc. Psychol..

[46] Harris D.J., Wilson M.R., Crowe E.M., Vine S.J. (2019). Examining the roles of working memory and visual attention in multiple object tracking expertise. Cogn. Process..

[47] Engle R.W. (2010). Role of working-memory capacity in cognitive control. Curr. Anthropol..

[48] Wood G., Vine S.J., Wilson M.R. (2016). Working memory capacity, controlled attention and aiming performance under pressure. Psychol. Res..

[49] Kreitz C., Furley P., Memmert D., Simons D.J. (2015). The Influence of Attention Set, Working Memory Capacity, and Expectations on Inattentional Blindness. Perception.

[50] Beilock S.L., Carr T.H. (2001). On the fragility of skilled performance: What governs choking under pressure?. J. Exp. Psychol. Gen..

[51] Bherer L., Kramer A.F., Peterson M.S., Colcombe S., Erickson K., Becic E. (2008). Transfer effects in task-set cost and dual-task cost after dual-task training in older and younger adults: Further evidence for cognitive plasticity in attentional control in late adulthood. Exp. Aging Res..

[52] Loffing F., Cañal-Bruland R. (2017). Anticipation in sport. Curr. Opin. Psychol..

